# Robot-assisted surgery: an emerging platform for human neuroscience research

**DOI:** 10.3389/fnhum.2015.00315

**Published:** 2015-06-04

**Authors:** Anthony M. Jarc, Ilana Nisky

**Affiliations:** ^1^Medical Research, Intuitive Surgical, Inc.Sunnyvale, CA, USA; ^2^Biomedical Engineering, Ben-Gurion University of the NegevBeer Sheva, Israel

**Keywords:** robot-assisted surgery, motor learning, sensorimotor control, robotics, control of movement, sensory integration, teleoperation, human-robot interaction

## Abstract

Classic studies in human sensorimotor control use simplified tasks to uncover fundamental control strategies employed by the nervous system. Such simple tasks are critical for isolating specific features of motor, sensory, or cognitive processes, and for inferring causality between these features and observed behavioral changes. However, it remains unclear how these theories translate to complex sensorimotor tasks or to natural behaviors. Part of the difficulty in performing such experiments has been the lack of appropriate tools for measuring complex motor skills in real-world contexts. Robot-assisted surgery (RAS) provides an opportunity to overcome these challenges by enabling unobtrusive measurements of user behavior. In addition, a continuum of tasks with varying complexity—from simple tasks such as those in classic studies to highly complex tasks such as a surgical procedure—can be studied using RAS platforms. Finally, RAS includes a diverse participant population of inexperienced users all the way to expert surgeons. In this perspective, we illustrate how the characteristics of RAS systems make them compelling platforms to extend many theories in human neuroscience, as well as, to develop new theories altogether.

## Introduction

**Figure 1:**
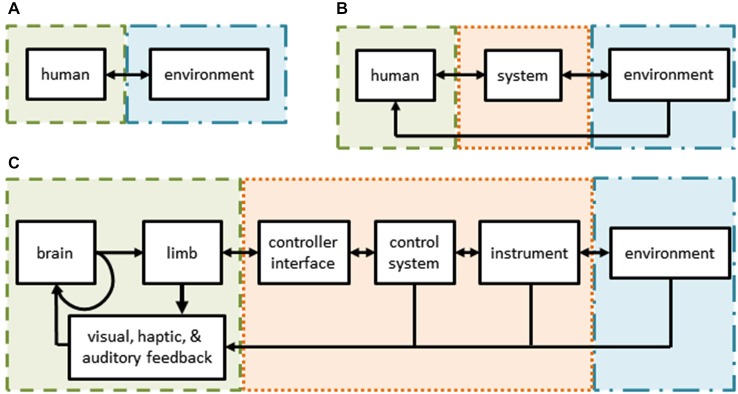
**(A)** Schematic of normal human interactions with the environment—the subject (green) interacts directly with her environment (blue) through sensorimotor channels. **(B)** Schematic of interactions in user-in-the-loop systems—the subject’s actions and/or senses are augmented by a control system and/or tools (orange) while interacting with her environment. **(C)** Schematic of robot assisted surgery—a form of a tele-operative (user-in-the-loop) system. The surgeon’s sensorimotor system is intimately tied to the teleoperative system through the controls, tools, and feedback modalities.

Humans are capable of exquisite behaviors. We move our bodies and seamlessly interact with tools and our environment to achieve desired outcomes. In general, humans generate motor commands, sense their actions and the environment, and estimate their internal state when trying to achieve a desired task (Figure 1A). Understanding such human behavior is essential to combating disease and injury and may be beneficial for designing systems with physical human-robot interactions.

However, it is very challenging to understand human behavior in natural environments. As a consequence, to uncover fundamental theories, researchers have developed techniques and methods to study human sensorimotor control that use basic tasks, such as: point-to-point reaching movements (Morasso, 1981; Flash and Hogan, 1985; Shadmehr and Mussa-Ivaldi, 1994; Krakauer et al., 2000; Shadmehr and Wise, 2005), reversal movements (Scheidt and Ghez, 2007), via-point movements (Flash and Hogan, 1985; Flash et al., 2013), drawing predefined shapes (Flash et al., 2013), manipulation of objects (Dingwell et al., 2002; Svinin et al., 2005; Leib and Karniel, 2012; Nasseroleslami and Sternad, 2014), throwing objects (Cohen and Sternad, 2009), lifting objects (Johansson and Flanagan, 2009; Mawase and Karniel, 2010), and bimanual reaches (Diedrichsen et al., 2010). To measure movements, robotic devices or other sensors may be used. Using a robotic device particularly advantageous because the robot can simultaneously measure movements and apply forces to the arm of the user as part of an experimental perturbation plan (Shadmehr and Mussa-Ivaldi, 1994; Karniel and Mussa-Ivaldi, 2002; Diedrichsen et al., 2005; Lackner and Dizio, 2005). However, adding a robotic device changes how the human interacts with his environment (Figure 1B). In this user-in-the-loop setting, a human’s actions are filtered by the robotic device, affected by its dynamics, and possibly controlled by different strategies altogether (Desmurget et al., 1997). In addition, virtual or augmented environments are often used to explore the response of the sensorimotor system to artificial modifications. Several examples include visuomotor rotations (Krakauer et al., 2000), force perturbations (Shadmehr and Mussa-Ivaldi, 1994), or delayed feedback (Pressman et al., 2007; Nisky et al., 2008). Additional, comprehensive reviews of state-of-the-art studies on sensorimotor control and learning can be found elsewhere (Shadmehr and Wise, 2005; Krakauer and Mazzoni, 2011; Shadmehr and Mussa-Ivaldi, 2012; Sigrist et al., 2013; Leukel et al., 2015).

Despite revealing many characteristics of the human sensorimotor system, these studies remain distant from representing natural behaviors during complex tasks (Wolpert et al., 2011). The movements in these studies are simple or abstract (although they may be building blocks of more complex movements Mussa-Ivaldi et al., 1994; Mussa-Ivaldi and Bizzi, 2000; Tresch and Jarc, 2009). Furthermore, users optimize these movements over hundreds of trials whereas natural behaviors take months, years, or a lifetime to master.

Complementary to basic research, more natural behaviors such as cello bowing (Verrel et al., 2013), stone knapping (Rein et al., 2013), tool-making (Faisal et al., 2010), golf swinging (Glazier, 2011), or baseball pitching (Chaisanguanthum et al., 2014) have been studied. One primary challenge with these studies is unobtrusively measuring subject behavior in their normal environment. Often, the sensors can adversely affect the behavior or fail to capture sufficient information. In addition, it can be difficult to draw strong parallels between studies of natural behaviors and studies that use abstract tasks.

Human neuroscience research would benefit from an experimental platform that: (1) spans basic to complex tasks; (2) extends to real-world applications; and (3) includes users of different levels of expertize. With such a platform, theories generated under basic conditions could be examined as task complexity increases to determine where and how they might break down or where new theories emerge (Fernandes and Kording, 2010). Furthermore, user populations with diverse levels of skill make it possible to examine learning during short training sessions (i.e., tens to hundreds of trials) or over prolonged timescales (Ericsson, 2004; Leukel et al., 2015).

**Figure 2:**
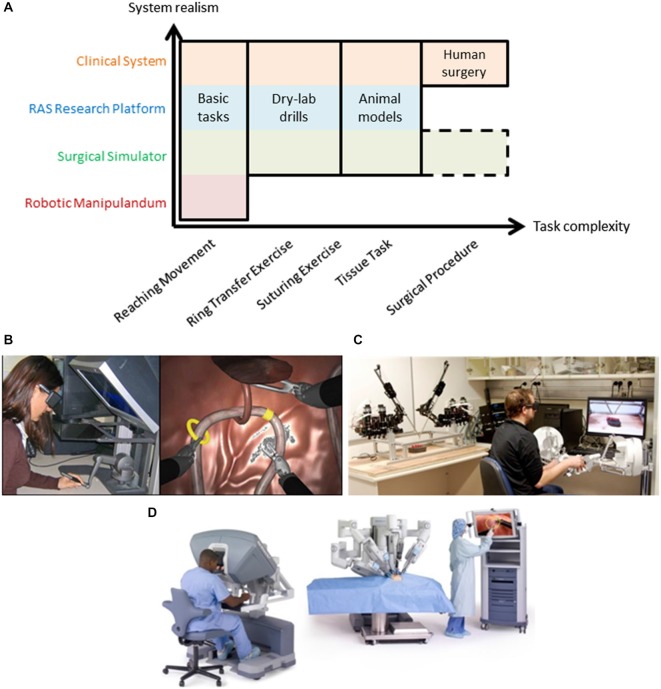
**(A)** Robot-assisted surgery offers a common test platform to study human sensorimotor control across many degrees of task complexity. Typical motor learning tasks utilize robotic manipulanda and basic tasks to understand the nervous system that cannot easily extend to more complex tasks (note the void in the bottom right of the figure). Various types of RAS systems offer increased exposure to more complex tasks while remaining suitable for many basic tasks, including clinical systems which can extend fully into complex human surgery (see top row in orange that spans task complexity). Note that surgical simulation extends to complex tasks (surgical procedures) but this remains an active research area (dashed outline). Examples of a RAS simulator **(B)**, research platform **(C)**, and clinical system **(D)** are shown. Note: Image from www.intuitivesurgical.com.

Here, we seek to highlight robot-assisted surgery (RAS) as a promising experimental platform for basic neuroscience research, as well as, applied clinical and technical research. RAS is a teleoperated system and its user interface is similar to many setups used for conventional motor learning and adaptation studies (Figures 1C, 2D). Importantly, RAS meets the three main objectives to serve as a useful experimental platform—it encompasses many levels of task complexity, system realism, and user expertize.

## RAS as an Experimental Platform

RAS is a widely used technology with thousands of surgeons performing operations each year (over 500,000 annual procedures using the *da Vinci*® Surgical System (Intuitive Surgical, Inc., Sunnyvale, CA) alone). The success of RAS as well as other new, emerging technologies is grounded in their ability to treat patients safely and effectively while reducing invasiveness. Given that a surgeon interacts with a robot to perform surgery, compelling opportunities exist for neuroscientists not only to advance our understanding of sensorimotor behavior, but also to improve RAS technology, surgeon training paradigms, and, ultimately, the experiences of patients who are treated with RAS.

During RAS, a surgeon sits at a console, views the operative field in three-dimensions, and uses master manipulators to control instruments inside a patient. The components of RAS are illustrated in Figure 1C. The surgeon’s motor system generates commands which cause hand movements (along with an efference copy). Her hands interact with master manipulators which serve as the input to the teleoperation system that controls the instruments or an endoscope held by a robotic arm. The instruments or endoscope then interact with the environment (i.e., the tissue of the patient). At each step, the surgeon senses various aspects of her behavior and the robotic system using visual, haptic, and auditory information channels. These, in turn, are combined with internal representations of the environment and efference copies to provide the surgeon with state estimates to drive subsequent actions, including online corrections and new movements, as well as to make strategic decisions about the upcoming steps of the surgical procedure. For example, to repair cancerous lymph nodes, a surgeon uses instruments to carefully palpate, dissect, and remove unhealthy tissue without damaging nearby structures.

RAS includes multiple parallel feedback loops around the motor system of the surgeon (Figure 1C). Each of these parallel loops is an opportunity to measure surgeon behavior or to apply structured perturbations. Many experimental paradigms require these measures or perturbations, such as cognitive reasoning, movement control, sensory processing, learning, and adaptation. During RAS, surgeon behavior can be measured at multiple stages, including hand movements, instrument movements, and the operative field of view. Similarly, perturbations can be applied to the surgeon by altering the movement or force applied to the master manipulators, the movements of the surgical instruments, or the visual feedback.

RAS research can be conducted using three classes of systems—simulators, research platforms, and clinical systems. As mentioned above, all of these classes meet three crucial objectives to serve as good experimental platforms for human neuroscience research—task complexity, system realism, and user expertize. Two of the three objectives are illustrated in Figure 2A. When compared to a robotic manipulandum, a common device for many neuroscience studies (see Shadmehr and Mussa-Ivaldi, 1994; Herzfeld et al., 2014; Wu et al., 2014b; Pekny and Shadmehr, 2015), all forms of RAS extend to more complex tasks (x-axis in Figure 2A) and have additional real-world applications (y-axis in Figure 2A). Here, we discuss each type of RAS system and its capacity to serve as a useful experimental platform for neuroscience research.

### RAS Simulators

RAS simulators can be simple haptic devices, replica systems (*dVTrainer*™, (Mimic Technologies, Inc., USA), *RobotiX* Mentor™ (3D Systems, Inc.), *RoSS*™ (Simulated Surgical Systems, LLC), etc.), or the actual surgeon console (*da Vinci Skills Simulator*™, Intuitive Surgical, Inc.) that all interface with a simulated environment (Figure 2B). The simulated environment can consist of abstract tasks (e.g., virtual dots for reaching tasks), simple tasks (e.g., ring transfer), or more complex tasks (e.g., suturing). Many of these tasks are formal training exercises used by surgeons to develop their skills prior to performing surgery (Stegemann et al., 2013; Smith et al., 2014). Recently, procedure-specific simulations have been created; however, this remains a research effort due to challenges simulating human tissue and its interactions with surgical tools (Cover et al., 1993; Misra et al., 2008, 2010; Jin et al., 2014). Evidence from validity studies of surgical simulations suggest high realism (McDougall, 2007; Kenney et al., 2009; Hung et al., 2011; Finnegan et al., 2012; Abboudi et al., 2013; Liss and McDougall, 2013), but realism tradeoffs are currently an open research question, and may depend on the fidelity of the simulation and the similarity of the master manipulator dynamics to the clinical system. Here, we place them just above classic, robotic manipulandum on the realism scale to highlight that an off-the-shelf haptic device may be converted into a surgical simulator if appropriate simulation software is used (Figure 2B; Coles et al., 2011; Ruthenbeck and Reynolds, 2013; Wu et al., 2014a).

### RAS Research Platforms

RAS research platforms are components of a surgical system (master manipulators and patient-side robotic arms) either designed for research, such as the RAVEN system that is depicted in Figure 2C. (Hannaford et al., 2013), or harvested from decommissioned clinical systems, such as the *daVinci* Research Kit, (Kazanzides et al., 2014). They aim to be flexible to meet needs of researchers from diverse areas of RAS. Here, we emphasize that RAS research platforms can be used for human neuroscience research during tasks of various complexities, excluding only live human surgery. The realism of these systems depends on the fidelity of the teleoperation controllers, but the behavior of the system may be configured to mimic the clinical system or entirely different experimental designs. For example, constraints may be imposed experimentally to elicit a smooth transition between classic studies and complex scenarios, such as initially constraining the master manipulator to two-dimensional movement.

### Clinical RAS Systems

Clinical systems are used by surgeons to perform surgery on patients (i.e., *da Vinci Si* Surgical System; Figure 2D). Although less flexible, they do offer levels of access through an application program interface on the system (DiMaio and Hasser, 2008), or equipping the system with external sensors for recording the master manipulator movements (Nisky et al., 2014a,c) or the patient-side manipulator movements (Tausch et al., 2012). Clinical systems can be configured for simple tasks (e.g., perform a dry-lab exercise; Jarc and Curet, 2014) while recording the same data during surgery. The powerful aspect of clinical systems is continuity—surgeons operate the same device for all types of tasks.

RAS platforms extend the continuum of task complexity beyond what is possible with robotic manipulanda. Also, they offer multiple levels of system realism from virtual environments to real-world tasks. In addition to task complexity and system realism, RAS platforms offer a subject pool that spans non-surgical persons unfamiliar with the technology to actual surgeons. The surgeon population consists of novices just beginning to use RAS all the way to experts who perform hundreds of cases per year. While an objective assessment of where along the learning curve a particular participant belongs is difficult (Ericsson, 2004), the immense subject pool offers unique opportunities to study many features of human neuroscience that might be challenging on other platforms (e.g., the characteristics of movement variability of novice surgeons over their first one hundred surgeries).

## Relevant RAS Research

In this section, we review several research areas within RAS that either directly relate to human neuroscience or motivate future studies.

### Examining Human Movement Control During RAS

A thorough understanding of how surgeons coordinate movement during RAS may reveal interesting aspects of sensorimotor control, as well, as provide a foundation to improve RAS technology. As a first step towards studying human movement control in RAS, an experimental setup was developed to compare simple planar point-to-point movements and freehand movements of experienced surgeons and novice, non-medical users tele-operating a clinical *da Vinci Si* Surgical System (Nisky et al., 2014a, c). The results from one study showed direction-dependent effects of tele-operation and level of expertize on several characteristics of user motion, including target acquisition error, movement speed, and movement smoothness (Nisky et al., 2014c). These effects may be explained via a dynamical model comprising the robotic manipulator, the arm of the surgeon, and the control strategy employed by the surgeon’s motor system, and may be adapted to within a single experimental session consisting of several hundred movements. A second study used the Uncontrolled Manifold framework (Latash et al., 2007; Scholz and Schöner, 2014) to demonstrate that experienced surgeons coordinated the variability of their joint angles to stabilize hand movements more than novice surgeons, especially during teleoperation (Nisky et al., 2014a). These results are consistent with many recent studies that suggest the motor system exploits redundancy to structure motor variability to maximize performance while minimizing control effort (Todorov and Jordan, 2002; Müller and Sternad, 2004; Cusumano and Cesari, 2006; Latash et al., 2007; Dingwell et al., 2013; Scholz and Schöner, 2014). Similarly, the results are consistent with studies suggesting that the ability to exploit redundancy is related to skill (Müller and Sternad, 2004; Cohen and Sternad, 2009) and task (Dingwell et al., 2013) or tool dynamics (Yang et al., 2007).

### Multisensory Integration on RAS Research Platforms

A surgeon must integrate information from multiple sensory channels in order to operate successfully using a RAS system. In one study, researchers showed that active use of a virtual-robotic (non-surgical) tool changed the spatial modulation of the crossmodal congruency effects (vision and touch multisensory integration), and that it did so in a manner comparable to changes in the representation of peripersonal space observed during real-world tool use (Sengül et al., 2012). In a second study, the same researchers showed that the crossmodal congruency effect was stronger after training with force feedback compared to without force feedback and when training with immediate force feedback compared to delayed force feedback (Sengül et al., 2013). The authors concluded that virtual surgical tool-use training with high-fidelity force feedback facilitated multisensory integration of signals from the tool, and hence embodiment of the tool.

### Surgeon Performance Enhancement Through Augmented Sensory Feedback

Current clinical RAS systems lack force feedback, and therefore surgeons rely primarily on visual information. However, the types of feedback that should be delivered to the user and during which tasks remain unknown. Engineering solutions will require thorough evaluation of surgeon behavior as additional sensory modalities are added to RAS systems. One research team has been examining how instrument vibrations could be harnessed and displayed to the user either through auditory or haptic feedback during RAS (McMahan et al., 2011; Bark et al., 2013; Koehn and Kuchenbecker, 2014). A recent study shows that both surgeons and non-surgeons prefer receiving feedback of instrument vibrations (Koehn and Kuchenbecker, 2014). Interestingly, the subjects were divided roughly equally in terms of whether they preferred haptic feedback alone or haptic and audio feedback. Despite this preference, the literature is inconclusive about performance differences with and without feedback (Okamura, 2009; Weber and Schneider, 2014). It remains an open research question as to how force feedback might influence a surgeon’s performance, and which aspects of force information might be critical to the surgeon. One might hypothesize that force feedback contributes to forming more accurate models of interactions with the external environment. For example, the adjustment of grip force that our fingers apply on hand-held tools in anticipation of the force that the environment applies on the tool depends on having access to force information (Danion et al., 2013; Gibo et al., 2014) or possibly how the tool is incorporated into a user’s internal model (Imamizu et al., 2000, 2003). Force feedback may also influence how and in which coordinate frames these internal models are represented, and consequently, how adaptation to novel environments generalizes (Shadmehr and Mussa-Ivaldi, 1994; Gandolfo et al., 1996; Krakauer et al., 2000; Shadmehr and Moussavi, 2000; Brayanov et al., 2012; Berniker et al., 2014; Rotella et al., 2015).

### Surgeon Skill Classification

Surgeon movement data and instrument movement and force data during dry-lab tasks have been used extensively to quantify the performance of surgeons. One particular approach attempted to create a “language of surgery” by decomposing surgeon movement into gestures called surgemes that could serve as fundamental building blocks to more complex behaviors (Lin et al., 2006). Note that surgemes strongly parallel the idea of motor primitives (Mussa-Ivaldi et al., 1994; Mussa-Ivaldi and Bizzi, 2000; Flash and Hochner, 2005). Other studies used stochastic models (Megali et al., 2006; Rosen et al., 2006) and movement trajectories (Judkins et al., 2009; Hofstad et al., 2013; Lendvay et al., 2013) to classify a surgeon’s skill. In an effort to encourage collaborations and idea generation, a surgical activity dataset was made publicly available online [the JHU-ISI Gesture and Skill Assessment Working Set (JIGSAWS; Gao et al., 2014)]. The dataset consists of kinematic data, video data, and manual annotations for eight surgeons with different levels of skill performing five repetitions of three elementary surgical tasks on a bench-top model using a *da Vinci* Surgical System. This dataset could be useful for neuroscientists to begin to propose new hypotheses and research studies related to human sensorimotor behavior in more real-world settings, and complement other publically available datasets such as the DREAM data set for reach movements (Walker and Kording, 2013), the stiffness probing dataset (Nisky et al., 2014b) and the gestures dataset (Frolova et al., 2013). Furthermore, such data sharing initiatives are imperative to research related to RAS since they enable researchers to begin exploring questions without requiring an experimental setup or a clinical system.

## Research Opportunities at the Intersection of RAS and Neuroscience

In summary, there exists an exciting opportunity to explore many research directions in human neuroscience by leveraging RAS as an experimental platform. We highlighted several relevant studies that spanned basic and applied research. Additional research areas include eye-hand coordination (Mylonas et al., 2008; Yang et al., 2008; Ahmidi et al., 2010), augmenting haptic, visual, or auditory channels to drive learning (Reinkensmeyer and Patton, 2009; Klein et al., 2012), or simplifying strategies during fine motor tasks (Tresch and Jarc, 2009).

Furthermore, human neuroscience research using RAS platforms could benefit from the existing user community. Engineering teams explore new platforms, instruments, and control features. Others develop new imaging modalities while still others examine user experience and human factors as they relate to RAS systems. Moreover, the clinical community evaluates efficacy, safety, cost, and surgeon training as they relate to RAS. Productive collaborations could be established between these research groups and those interested in human neuroscience, especially since the surgeon influences the final system behavior (assuming non-autonomous, user-in-the-loop setups). To foster collaborations, routine workshops are being held for users of RAS research platforms.

## Limitations of Using RAS as an Experimental Platform

Although many compelling aspects of RAS were outlined, several potential limitations and challenges to using RAS for human neuroscience research exist. Firstly, the availability of RAS systems is limited. For RAS research platforms, one solution could be to have multiple research groups at an institution share the equipment. For clinical RAS systems, strong collaborations with clinical researchers and surgeons would improve the likelihood of access to systems when they are not being used for surgeries. Secondly, the cost of RAS research platforms is significant. Despite this, many interesting questions can be explored using simpler setups, such as inexpensive and accessible haptic devices equipped with virtual reality simulators, before being translated to RAS research platforms (Figure 2B). Furthermore, the costs will likely decrease as the user community grows. A third limitation could be the RAS system properties. For example, the master manipulators may not have adequate stiffness for certain experimental conditions or require unnatural subject interfaces. Finally, the growth of shared datasets and RAS research platforms remain moderate. Growth can be accelerated with interest from more research teams, which, in turn, would enable additional researchers to leverage RAS systems for their studies. Given these limitations, alternative platforms outside of RAS, such as gaming consoles and flight simulators, may also be useful research tools for understanding sensorimotor control. Similar to RAS, these platforms are real-life applications with users of a wide range of ability. Interesting insights could result from comparisons between these platforms and RAS.

## Conclusions

In this perspective, we highlight the potential for RAS to become an influential experimental platform for human neuroscience research that bridges the gap between laboratory experiments and real-world applications. RAS offers the unique opportunity to examine how theories in human sensorimotor control evolve from abstract tasks to more complex behaviors using simulators, research platforms, or clinical systems. In the end, human neuroscience research that uses RAS platforms has the potential to improve the lives of individuals suffering from motor impairments as well as patients undergoing surgery for a variety of diseases.

## Conflict of Interest Statement

A. M. Jarc is a researcher in the Medical Research group at Intuitive Surgical, Inc. I. Nisky declares that the research was conducted in the absence of any commercial or financial relationships that could be construed as a potential conflict of interest.

## References

[B1] Abboudi H. Khan M. S. Aboumarzouk O. Guru K. A. Challacombe B. Dasgupta P.. (2013). Current status of validation for robotic surgery simulators—a systematic review. BJU Int. 111, 194–205. 10.1111/j.1464-410x.2012.11270.x 22672340

[B2] Ahmidi N. Hager G. D. Ishii L. Fichtinger G. Gallia G. L. Ishii M. (2010). Surgical task and skill classification from eye tracking and tool motion in minimally invasive surgery. Med. Image Comput. Comput. Assist. Interv. 6363, 295–302. 10.1007/978-3-642-15711-0_37 20879412

[B3] Bark K. Mcmahan W. Remington A. Gewirtz J. Wedmid A. Lee D. I.. (2013). *In vivo* validation of a system for haptic feedback of tool vibrations in robotic surgery. Surg. Endosc. 27, 656–664. 10.1007/s00464-012-2452-8 22806517

[B4] Berniker M. Franklin D. W. Flanagan J. R. Wolpert D. M. Kording K. (2014). Motor learning of novel dynamics is not represented in a single global coordinate system: evaluation of mixed coordinate representations and local learning. J. Neurophysiol. 111, 1165–1182. 10.1152/jn.00493.2013 24353296 PMC3949315

[B5] Brayanov J. B. Press D. Z. Smith M. A. (2012). Motor memory is encoded as a gain-field combination of intrinsic and extrinsic action representations. J. Neurosci. 32, 14951–14965. 10.1523/jneurosci.1928-12.2012 23100418 PMC3999415

[B6] Chaisanguanthum K. S. Shen H. H. Sabes P. N. (2014). Motor variability arises from a slow random walk in neural state. J. Neurosci. 34, 12071–12080. 10.1523/jneurosci.3001-13.2014 25186752 PMC4152607

[B7] Cohen R. Sternad D. (2009). Variability in motor learning: relocating, channeling and reducing noise. Exp. Brain Res. 193, 69–83. 10.1007/s00221-008-1596-1 18953531 PMC2756422

[B8] Coles T. R. Meglan D. John N. (2011). The role of haptics in medical training simulators: a survey of the state of the art. IEEE Trans. Haptics 4, 51–66. 10.1109/toh.2010.19 26962955

[B9] Cover S. A. Ezquerra N. F. O’brien J. F. Rowe R. Gadacz T. Palm E. (1993). Interactively deformable models for surgery simulation. IEEE Comput. Graph. Appl. 13, 68–75. 10.1109/38.252559

[B10] Cusumano J. P. Cesari P. (2006). Body-goal variability mapping in an aiming task. Biol. Cybern. 94, 367–379. 10.1007/s00422-006-0052-1 16501988

[B11] Danion F. Diamond J. S. Flanagan J. R. (2013). Separate contributions of kinematic and kinetic errors to trajectory and grip force adaptation when transporting novel hand-held loads. J. Neurosci. 33, 2229–2236. 10.1523/jneurosci.3772-12.2013 23365258 PMC6619110

[B12] Desmurget M. Jordan M. Prablanc C. Jeannerod M. (1997). Constrained and unconstrained movements involve different control strategies. J. Neurophysiol. 77, 1644–1650. 9084629 10.1152/jn.1997.77.3.1644

[B13] Diedrichsen J. Hashambhoy Y. Rane T. Shadmehr R. (2005). Neural correlates of reach errors. J. Neurosci. 25, 9919–9931. 10.1523/jneurosci.1874-05.2005 16251440 PMC1479774

[B14] Diedrichsen J. Shadmehr R. Ivry R. B. (2010). The coordination of movement: optimal feedback control and beyond. Trends Cogn. Sci. 14, 31–39. 10.1016/j.tics.2009.11.004 20005767 PMC4350769

[B15] DiMaio S. Hasser C. (2008). “The da Vinci research interface,” in MICCAI Workshop on Systems and Arch. for Computer Assisted Interventions, Midas Journal. Available online at: http://hdl.handle.net/10380/1464

[B16] Dingwell J. B. Mah C. D. Mussa-Ivaldi F. A. (2002). Manipulating objects with internal degrees of freedom: evidence for model-based control. J. Neurophysiol. 88, 222–235. 10.1152/jn00454.2001 12091548

[B17] Dingwell J. B. Smallwood R. F. Cusumano J. P. (2013). Trial-to-trial dynamics and learning in a generalized, redundant reaching task. J. Neurophysiol. 109, 225–237. 10.1152/jn.00951.2011 23054607 PMC3545167

[B18] Ericsson K. A. (2004). Deliberate practice and the acquisition and maintenance of expert performance in medicine and related domains. Acad. Med. 79, S70–S81. 10.1097/00001888-200410001-00022 15383395

[B19] Faisal A. Stout D. Apel J. Bradley B. (2010). The manipulative complexity of lower paleolithic stone toolmaking. PLoS One 5:e13718. 10.1371/journal.pone.0013718 21072164 PMC2972205

[B20] Fernandes H. L. Kording K. P. (2010). In praise of “false” models and rich data. J. Mot. Behav. 42, 343–349. 10.1080/00222895.2010.526462 21184351

[B21] Finnegan K. T. Meraney A. M. Staff I. Shichman S. J. (2012). da Vinci skills simulator construct validation study: correlation of prior robotic experience with overall score and time score simulator performance. Urology 80, 330–336. 10.1016/j.urology.2012.02.059 22704177

[B22] Flash T. Hochner B. (2005). Motor primitives in vertebrates and invertebrates. Curr. Opin. Neurobiol. 15, 660–666. 10.1016/j.conb.2005.10.011 16275056

[B23] Flash T. Hogan N. (1985). The coordination of arm movements: an experimentally confirmed mathematical model. J. Neurosci. 5, 1688–1703. 4020415 10.1523/JNEUROSCI.05-07-01688.1985 PMC6565116

[B24] Flash T. Meirovitch Y. Barliya A. (2013). Models of human movement: trajectory planning and inverse kinematics studies. Rob. Auton. Syst. 61, 330–339. 10.1016/j.robot.2012.09.020

[B25] Frolova D. Stern H. Berman S. (2013). Most probable longest common subsequence for recognition of gesture character input. IEEE Trans. Cybern. 43, 871–880. 10.1109/tsmcb.2012.2217324 23047881

[B26] Gandolfo F. Mussa-Ivaldi F. Bizzi E. (1996). Motor learning by field approximation. Proc. Natl. Acad. Sci. U S A 93, 3843–3846. 10.1073/pnas.93.9.3843 8632977 PMC39446

[B27] Gao Y. Vedula S. S. Reiley C. E. Ahmidi N. Varadarajan B. Lin H. C. (2014). “The JHU-ISI Gesture and Skill Assessment Working Set (JIGSAWS): a surgical activity dataset for human motion modeling,” in Modeling and Monitoring of Computer Assisted Interventions (M2CAI), MICCAI Workshop (Boston, MA).

[B28] Gibo T. L. Bastian A. J. Okamura A. M. (2014). Grip force control during virtual object interaction: effect of force feedback, accuracy demands and training. IEEE Trans. Haptics 7, 37–47. 10.1109/TOH.2013.60 24845744

[B29] Glazier P. (2011). Movement variability in the golf swing: theoretical, methodological and practical issues. Res. Q. Exerc. Sport 82, 157–161. 10.5641/027013611x13119541883429 21699094

[B30] Hannaford B. Rosen J. Friedman D. W. King H. Roan P. Cheng L.. (2013). Raven-II: an open platform for surgical robotics research. IEEE Trans. Biomed. Eng. 60, 954–959. 10.1109/TBME.2012.2228858 23204264

[B31] Herzfeld D. J. Vaswani P. A. Marko M. K. Shadmehr R. (2014). A memory of errors in sensorimotor learning. Science 345, 1349–1353. 10.1126/science.1253138 25123484 PMC4506639

[B32] Hofstad E. F. Våpenstad C. Chmarra M. K. Langø T. Kuhry E. Mårvik R. (2013). A study of psychomotor skills in minimally invasive surgery: what differentiates expert and nonexpert performance. Surg. Endosc. 27, 854–863. 10.1007/s00464-012-2524-9 23052505

[B33] Hung A. J. Zehnder P. Patil M. B. Cai J. Ng C. K. Aron M.. (2011). Face, content and construct validity of a novel robotic surgery simulator. J. Urol. 186, 1019–1025. 10.1016/j.juro.2011.04.064 21784469

[B34] Imamizu H. Kuroda T. Miyauchi S. Yoshioka T. Kawato M. (2003). Modular organization of internal models of tools in the human cerebellum. Proc. Natl. Acad. Sci. U S A 100, 5461–5466. 10.1073/pnas.0835746100 12704240 PMC154367

[B35] Imamizu H. Miyauchi S. Tamada T. Sasaki Y. Takino R. Puètz B.. (2000). Human cerebellar activity reflecting an acquired internal model of a new tool. Nature 403, 192–195. 10.1038/35003194 10646603

[B36] Jarc A. M. Curet M. (2014). Construct validity of nine new inanimate exercises for robotic surgeon training using a standardized setup. Surg. Endosc. 28, 648–656. 10.1007/s00464-013-3224-9 24100861

[B37] Jin X. Joldes G. R. Miller K. Yang K. H. Wittek A. (2014). Meshless algorithm for soft tissue cutting in surgical simulation. Comput. Methods Biomech. Biomed. Engin. 17, 800–811. 10.1080/10255842.2012.716829 22974246

[B38] Johansson R. S. Flanagan J. R. (2009). Coding and use of tactile signals from the fingertips in object manipulation tasks. Nat. Rev. Neurosci. 10, 345–359. 10.1038/nrn2621 19352402

[B39] Judkins T. N. Oleynikov D. Stergiou N. (2009). Objective evaluation of expert and novice performance during robotic surgical training tasks. Surg. Endosc. 23, 590–597. 10.1007/s00464-008-9933-9 18443870

[B40] Karniel A. Mussa-Ivaldi F. (2002). Does the motor control system use multiple models and context switching to cope with a variable environment? Exp. Brain Res. 143, 520–524. 10.1007/s00221-002-1054-4 11914799

[B41] Kazanzides P. Chen Z. Deguet A. Fischer G. Taylor R. Dimaio S. (2014). “An open-source research kit for the da Vinci R surgical robot,” in Robotics and Automation (ICRA), 2014 IEEE International Conference on (Hong Kong).

[B42] Kenney P. A. Wszolek M. F. Gould J. J. Libertino J. A. Moinzadeh A. (2009). Face, content and construct validity of dV-trainer, a novel virtual reality simulator for robotic surgery. Urology 73, 1288–1292. 10.1016/j.urology.2008.12.044 19362352

[B43] Klein J. Spencer S. J. Reinkensmeyer D. J. (2012). Breaking it down is better: haptic decomposition of complex movements aids in robot-assisted motor learning. IEEE Trans. Neural Syst. Rehabil. Eng. 20, 268–275. 10.1109/TNSRE.2012.2195202 22531825 PMC4015469

[B44] Koehn J. K. Kuchenbecker K. J. (2014). Surgeons and non-surgeons prefer haptic feedback of instrument vibrations during robotic surgery. Surg. Endosc. [Epub ahead of print]. 10.1007/s00464-014-4030-8 25539693

[B45] Krakauer J. W. Mazzoni P. (2011). Human sensorimotor learning: adaptation, skill and beyond. Curr. Opin. Neurobiol. 21, 636–644. 10.1016/j.conb.2011.06.012 21764294

[B46] Krakauer J. W. Pine Z. M. Ghilardi M.-F. Ghez C. (2000). Learning of visuomotor transformations for vectorial planning of reaching trajectories. J. Neurosci. 20, 8916–8924. 11102502 10.1523/JNEUROSCI.20-23-08916.2000 PMC6773094

[B47] Lackner J. R. Dizio P. (2005). Motor control and learning in altered dynamic environments. Curr. Opin. Neurobiol. 15, 653–659. 10.1016/j.conb.2005.10.012 16271464

[B48] Latash M. L. Scholz J. P. Schöner G. (2007). Toward a new theory of motor synergies. Motor Control 11, 276–308. 17715460 10.1123/mcj.11.3.276

[B49] Leib R. Karniel A. (2012). Minimum acceleration with constraints of center of mass: a unified model for arm movements and object manipulation. J. Neurophysiol. 108, 1646–1655. 10.1152/jn.00224.2012 22696546

[B50] Lendvay T. S. Brand T. C. White L. Kowalewski T. Jonnadula S. Mercer L. D.. (2013). Virtual reality robotic surgery warm-up improves task performance in a dry laboratory environment: a prospective randomized controlled study. J. Am. Coll. Surg. 216, 1181–1192. 10.1016/j.jamcollsurg.2013.02.012 23583618 PMC4082669

[B51] Leukel C. Gollhofer A. Taube W. (2015). In experts, underlying processes that drive visuomotor adaptation are different than in novices. Front. Hum. Neurosci. 9:50. 10.3389/fnhum.2015.00050 25713526 PMC4322639

[B52] Lin H. C. Shafran I. Yuh D. Hager G. D. (2006). Towards automatic skill evaluation: detection and segmentation of robot-assisted surgical motions. Comput. Aided Surg. 11, 220–230. 10.3109/10929080600989189 17127647

[B53] Liss M. A. McDougall E. M. (2013). Robotic surgical simulation. Cancer J. 19, 124–129. 10.1097/PPO.0b013e3182885d79 23528719

[B54] Mawase F. Karniel A. (2010). Evidence for predictive control in lifting series of virtual objects. Exp. Brain Res. 203, 447–452. 10.1007/s00221-010-2249-8 20428856

[B55] McDougall E. M. (2007). Validation of surgical simulators. J. Endourol. 21, 244–247. 10.1089/end.2007.9985 17444766

[B56] McMahan W. Gewirtz J. Standish D. Martin P. Kunkel J. A. Lilavois M. (2011). Tool contact acceleration feedback for telerobotic surgery. IEEE Trans. Haptics 4, 210–220. 10.1109/toh.2011.31 26963488

[B57] Megali G. Sinigaglia S. Tonet O. Dario P. (2006). Modelling and evaluation of surgical performance using hidden Markov models. IEEE Trans. Biomed. Eng. 53, 1911–1919. 10.1109/tbme.2006.881784 17019854

[B58] Misra S. Ramesh K. Okamura A. (2008). Modeling of tool-tissue interactions for computer-based surgical simulation: a literature review. Presence (Camb.) 17, 463–491. 10.1162/pres.17.5.463 20119508 PMC2813063

[B59] Misra S. Ramesh K. Okamura A. M. (2010). Modelling of non-linear elastic tissues for surgical simulation. Comput. Methods Biomech. Biomed. Engin. 13, 811–818. 10.1080/10255840903505121 20503126 PMC3050496

[B60] Morasso P. (1981). Spatial control of arm movements. Exp. Brain Res. 42, 223–227. 10.1007/bf00236911 7262217

[B61] Müller H. Sternad D. (2004). Decomposition of variability in the execution of goal-oriented tasks: three components of skill improvement. J. Exp. Psychol. Hum. Percept. Perform. 30, 212–233. 10.1037/0096-1523.30.1.212 14769078

[B62] Mussa-Ivaldi F. A. Bizzi E. (2000). Motor learning through the combination of primitives. Philos. Trans. R. Soc. Lond. B Biol. Sci. 355, 1755–1769. 10.1098/rstb.2000.0733 11205339 PMC1692905

[B63] Mussa-Ivaldi F. A. Giszter S. F. Bizzi E. (1994). Linear combinations of primitives in vertebrate motor control. Proc. Natl. Acad. Sci. U S A 91, 7534–7538. 10.1073/pnas.91.16.7534 8052615 PMC44436

[B64] Mylonas G. P. Kwok K.-W. Darzi A. Yang G.-Z. (2008). Gaze-contingent motor channelling and haptic constraints for minimally invasive robotic surgery. Med. Image Comput. Comput. Assist. Interv. 11, 676–683. 10.1007/978-3-540-85990-1_81 18982663

[B65] Nasseroleslami B. Sternad D. (2014). “Extrinsic contributions to movement variability in human object manipulation,” in 40th Annual Northeast Bioengineering Conference (NEBEC) (Boston, MA), 1–2.

[B66] Nisky I. Hsieh M. Okamura A. (2014a). Uncontrolled manifold analysis of arm joint angle variability during robotic teleoperation and freehand movement of surgeons and novices. IEEE Trans. Biomed. Eng. 61, 2869–2881. 10.1109/TBME.2014.2332359 24967980 PMC8085739

[B67] Nisky I. Leib R. Milstein A. Karniel A. (2014b). “Perception of stiffness with force feedback delay,” in Multisensory Softness (London: Springer), 167–185.

[B68] Nisky I. Mussa-Ivaldi F. A. Karniel A. (2008). A regression and boundary-crossing-based model for the perception of delayed stiffness. IEEE Trans. Haptics 1, 73–82. 10.1109/toh.2008.17 27780151

[B69] Nisky I. Okamura A. M. Hsieh M. H. (2014c). Effects of robotic manipulators on movements of novices and surgeons. Surg. Endosc. 28, 2145–2158. 10.1007/s00464-014-3446-5 24519031 PMC8101070

[B70] Okamura A. M. (2009). Haptic feedback in robot-assisted minimally invasive surgery. Curr. Opin. Urol. 19, 102–107. 10.1097/mou.0b013e32831a478c 19057225 PMC2701448

[B71] Pekny S. E. Shadmehr R. (2015). Optimizing effort: increased efficiency of motor memory with time away from practice. J. Neurophysiol. 113, 445–454. 10.1152/jn.00638.2014 25355964 PMC4297790

[B72] Pressman A. Welty L. J. Karniel A. Mussa-Ivaldi F. A. (2007). Perception of delayed stiffness. Int. J. Rob. Res. 26, 1191–1203. 10.1177/0278364907082611

[B73] Rein R. Bril B. Nonaka T. (2013). Coordination strategies used in stone knapping. Am. J. Phys. Anthropol. 150, 539–550. 10.1002/ajpa.22224 23359287

[B74] Reinkensmeyer D. J. Patton J. L. (2009). Can robots help the learning of skilled actions? Exerc. Sport Sci. Rev. 37, 43–51. 10.1097/JES.0b013e3181912108 19098524 PMC2905644

[B75] Rosen J. Brown J. D. Chang L. Sinanan M. N. Hannaford B. (2006). Generalized approach for modeling minimally invasive surgery as a stochastic process using a discrete Markov model. IEEE Trans. Biomed. Eng. 53, 399–413. 10.1109/tbme.2005.869771 16532766

[B76] Rotella M. F. Nisky I. Koehler M. Rinderknecht M. D. Bastian A. J. Okamura A. M. (2015). Learning and generalization in an isometric visuomotor task. J. Neurophysiol. 113, 1873–1884. 10.1152/jn.00255.2014 25520430

[B77] Ruthenbeck G. S. Reynolds K. J. (2013). Virtual reality surgical simulator software development tools. J. Simul. 7, 101–108. 10.1057/jos.2012.22

[B78] Scheidt R. A. Ghez C. (2007). Separate adaptive mechanisms for controlling trajectory and final position in reaching. J. Neurophysiol. 98, 3600–3613. 10.1152/jn.00121.2007 17913996

[B79] Scholz J. P. Schöner G. (2014). Use of the uncontrolled manifold (UCM) approach to understand motor variability, motor equivalence and self-motion. Adv. Exp. Med. Biol. 826, 91–100. 10.1007/978-1-4939-1338-1_7 25330887

[B80] Sengül A. Rognini G. van Elk M. Aspell J. E. Bleuler H. Blanke O. (2013). Force feedback facilitates multisensory integration during robotic tool use. Exp. Brain Res. 227, 497–507. 10.1007/s00221-013-3526-0 23625046

[B81] Sengül A. Van Elk M. Rognini G. Aspell J. E. Bleuler H. Blanke O. (2012). Extending the body to virtual tools using a robotic surgical interface: evidence from the crossmodal congruency task. PLoS One 7:e49473. 10.1371/journal.pone.0049473 23227142 PMC3515602

[B82] Shadmehr R. Moussavi Z. M. (2000). Spatial generalization from learning dynamics of reaching movements. J. Neurosci. 20, 7807–7815. 11027245 10.1523/JNEUROSCI.20-20-07807.2000 PMC6772893

[B83] Shadmehr R. Mussa-Ivaldi F. A. (1994). Adaptive representation of dynamics during learning of a motor task. J. Neurosci. 14, 3208–3224. 8182467 10.1523/JNEUROSCI.14-05-03208.1994 PMC6577492

[B84] Shadmehr R. Mussa-Ivaldi S. (2012). Biological Learning and Control: How the Brain Builds Representations, Predicts Events and Makes Decisions. Cambridge, MA: MIT Press.

[B85] Shadmehr R. Wise S. P. (2005). The Computational Neurobiology of Reaching and Pointing: A Foundation for Motor Learning. Computational Neuroscience. Cambridge, MA: MIT Press, xviii, 575.

[B86] Sigrist R. Rauter G. Riener R. Wolf P. (2013). Augmented visual, auditory, haptic and multimodal feedback in motor learning: a review. Psychon. Bull. Rev. 20, 21–53. 10.3758/s13423-012-0333-8 23132605

[B87] Smith R. Patel V. Satava R. (2014). Fundamentals of robotic surgery: a course of basic robotic surgery skills based upon a 14-society consensus template of outcomes measures and curriculum development. Int. J. Med. Robot. 10, 379–384. 10.1002/rcs.1559 24277315

[B88] Stegemann A. P. Ahmed K. Syed J. R. Rehman S. Ghani K. Autorino R.. (2013). Fundamental skills of robotic surgery: a multi-institutional randomized controlled trial for validation of a simulation-based curriculum. Urology 81, 767–774. 10.1016/j.urology.2012.12.033 23484743

[B89] Svinin M. Masui Y. Luo Z. W. Hosoe S. (2005). On the dynamic version of the minimum hand jerk criterion. J. Rob. Syst. 22, 661–676. 10.1002/rob.20091

[B90] Tausch T. J. Kowalewski T. M. White L. W. McDonough P. S. Brand T. C. Lendvay T. S. (2012). Content and construct validation of a robotic surgery curriculum using an electromagnetic instrument tracker. J. Urol. 188, 919–923. 10.1016/j.juro.2012.05.005 22819403

[B91] Todorov E. Jordan M. I. (2002). Optimal feedback control as a theory of motor coordination. Nat. Neurosci. 5, 1226–1235. 10.1038/nn963 12404008

[B92] Tresch M. C. Jarc A. (2009). The case for and against muscle synergies. Curr. Opin. Neurobiol. 19, 601–607. 10.1016/j.conb.2009.09.002 19828310 PMC2818278

[B93] Verrel J. Pologe S. Manselle W. Lindenberger U. Woollacott M. (2013). Coordination of degrees of freedom and stabilization of task variables in a complex motor skill: expertise-related differences in cello bowing. Exp. Brain Res. 224, 323–334. 10.1007/s00221-012-3314-2 23109087

[B94] Walker B. Kording K. (2013). The database for reaching experiments and models. PLoS One 8:e78747. 10.1371/journal.pone.0078747 24244351 PMC3828298

[B95] Weber B. Schneider S. (2014). “The effects of force feedback on surgical task performance: a meta-analytical integration,” in Haptics: Neuroscience, Devices, Modeling and Applications, eds Auvray M. Duriez C. (Heidelberg, Berlin: Springer), 150–157.

[B96] Wolpert D. M. Diedrichsen J. Flanagan J. R. (2011). Principles of sensorimotor learning. Nat. Rev. Neurosci. 12, 739–751. 10.1038/nrn3112 22033537

[B97] Wu F. Chen X. Lin Y. Wang C. Wang X. Shen G.. (2014a). A virtual training system for maxillofacial surgery using advanced haptic feedback and immersive workbench. Int. J. Med. Robot. 10, 78–87. 10.1002/rcs.1514 23720249

[B98] Wu H. G. Miyamoto Y. R. Castro L. N. G. Ölveczky B. P. Smith M. A. (2014b). Temporal structure of motor variability is dynamically regulated and predicts motor learning ability. Nat. Neurosci. 17, 312–321. 10.1038/nn.3616 24413700 PMC4442489

[B99] Yang G.-Z. Mylonas G. P. Kwok K.-W. Chung A. (2008). Perceptual docking for robotic control. Med. Imaging Augmented Real. 5128, 21–30. 10.1007/978-3-540-79982-5_3

[B100] Yang J.-F. Scholz J. P. Latash M. L. (2007). The role of kinematic redundancy in adaptation of reaching. Exp. Brain Res. 176, 54–69. 10.1007/s00221-006-0602-8 16874517 PMC1945250

